# Book Notices

**Published:** 1889-12

**Authors:** 


THE MEDICAL BULLETIN Visiting list or Physician's Call Record.—Arranged upon an Original and convenient monthly and weekly plan for the daily recording of professional visits. Philadelphia and London. F. A. Davis, Publisher.

Truly an exceedingly convenient and admirably arranged register and memo dum book for the practitioner.

A MANUAL OF DIETETICS for Physicians, mothers and nurses, by W. B. Pritchard, M. D., New York City. 83 pages in cloth. Published by the Dietetic Publishing Co., New York.

LILLY'S HAND BOOK OF PHARMACY AND THERAPEUTICS.—Third edition, thoroughly revised. Eli Lilly & Co., Indianapolis. 1888. pp. 246.

This work presents in neat and convenient form the numerous and excellent formulæ of the large and well known house of Eli Lilly & Co. It is not so much an advertisement of special preparations as a compilation from standard authorities, containing also posological table, abbreviations and index of diseases with remedies, etc.

A MANUAL OF INSTRUCTION for giving Sweedish movement and massage treatment, by Professor Hartwig Nissen, Director of the Sweedish Health Institute, Washington; Late Instructor in Physical Culture and Gymnastics, at the Johns Hopkins University Baltimore Md. Author of Health by Exercise Without Apparatus, with 29 original wood engravings. Philadelphia and London. F. A. Davis, Publisher. Price, $1.00 net.

An interesting work of 125 pages. Containing History; classification of movements; description of movements; the vibrator; diseases and their treatment.

Under the general groups are numerous interesting and practical details, in the study of which a fair understanding and practical knowledge of this interesting department of the healing art may be obtained.

ESTIMATES OF PATHOLOGY AND MORBID ANATOMY, by C. E. Armend Semple, A. B., M. D., Cautab. L. S. A., M. D., M. R. C. P., London, Physician to the Bloomsbury Dispensary; Physician to the North Eastern Hospital for children, etc., etc., with forty-six illustrations. Philadelphia, W. B. Saunders 913 Walnut st.; London, Henry Renshaw; Melbourne, George Robertson & Co., 1890. This is a condensed but comprehensive outline of *Inflammation* in all of its phases; *Tuberculosis*, *Degenerations*, *Tumors*. Teratomata, Septicaemia, Pyaemia, Hyperaemia, Ischaemia. Leucocythæmia, uraemia, Syphilis; Hypertrophy, Atrophy, Necrosis; Rachitis; Pathology of urine; Animal parasites; Vegitable parasites; Pathogenic bacilli; Blastomycetæ, Hypomecetae; Methods for recognizing bacteria, etc.

REPORT OF AMPUTATIONS performed at the Hospital of the University of Pennsylvania, from September 30, 1874, to December 31, 1888. By Lewis H. Adler, Jr., M. D., late resident physician, University Hospital.

A CASE OF HODGIN'S DISEASE, accompanied with a possible resulting paraplegia. Reported by Lewis H. Adler, Jr., M. D., Resident physician, University Hospital, Philadelphia.

REPORT of a case of hystero-epilepsy in a man, by Lewis H. Adler, Jr., M. D., Resident physician, University Hospital, Philadelphia.

REPORT of a case of stricture of the rectum, the probable result of a specific vaginitis. By Lewis H. Adler, Jr., M. D., late Resident physician, University Hospital, Philadelphia, Penn.

PHYSICIAN'S VISITING LIST.—Lindsay & Blakiston, for 1890. Thirty-ninth year of its publication. Philadelphia, P. Blakiston, Son & Co., 1012 Walnut St. One of the best of all the pocket ledgers printed. Containing calender, dose table, measures; examination of urine and numerous useful facts and memoranda.

